# ParaDeep: sequence-based deep learning for residue-level paratope prediction using chain-aware BiLSTM-CNN models

**DOI:** 10.3389/fbinf.2025.1684042

**Published:** 2025-11-05

**Authors:** Piyachat Udomwong, Thanathat Pamonsupornwichit, Kanchanok Kodchakorn, Chatchai Tayapiwatana

**Affiliations:** 1 International College of Digital Innovation, Chiang Mai University, Chiang Mai, Thailand; 2 Center of Biomolecular Therapy and Diagnostic, Faculty of Associated Medical Sciences, Chiang Mai University, Chiang Mai, Thailand; 3 Office of Research Administration, Chiang Mai University, Chiang Mai, Thailand; 4 Department of Chemistry, Faculty of Science, Chiang Mai University, Chiang Mai, Thailand; 5 Division of Clinical Immunology, Department of Medical Technology, Faculty of Associated Medical Sciences, Chiang Mai University, Chiang Mai, Thailand

**Keywords:** antibody binding site prediction, deep learning, BiLSTM-CNN, heavy and light chains, paratope identification, sequence modeling

## Abstract

Accurate prediction of antibody paratopes is a critical challenge in structure-limited, high-throughput discovery workflows. We present ParaDeep, a lightweight and interpretable deep learning framework for residue-level paratope prediction directly from amino acid sequences. ParaDeep integrates bidirectional long short-term memory networks with one-dimensional convolutional layers to capture both long-range sequence context and local binding motifs. We systematically evaluated 30 model configurations varying in encoding schemes, convolutional kernel sizes, and antibody chain types. In five-fold cross-validation, heavy (H) chain models achieved the highest performance (F1 = 0.856 ± 0.014, MCC = 0.842 ± 0.015), outperforming light (L) chain models (F1 = 0.774 ± 0.023, MCC = 0.772 ± 0.022). On an independent blind test set, ParaDeep attained F1 = 0.723 and MCC = 0.685 for H chains, and F1 = 0.607 and MCC = 0.587 for L chains, representing a 27% MCC improvement over the sequence-based baseline Parapred. Chain-specific modeling revealed that heavy chains provide stronger sequence-based predictive signals, while light chains benefit more from structural context. ParaDeep approaches the performance of state-of-the-art structure-based methods on heavy chains while requiring only sequence input, enabling faster and broader applicability without the computational cost of 3D modeling. Its efficiency and scalability make it well-suited for early-stage antibody discovery, repertoire profiling, and therapeutic design, particularly in the absence of structural data. The implementation is freely available at https://github.com/PiyachatU/ParaDeep, with Python (PyTorch) code and a Google Colab interface for ease of use.

## 1 Introduction

Antibodies neutralize antigens through a subset of surface-exposed residues known as paratopes (a set of antibody residues in direct contact with the antigen), which are predominantly located in the hypervariable loops within the variable domain of heavy (VH) and light (VL) chains, termed complementarity-determining regions (CDRs) (Chothia and Lesk, 1987; Foote and Winter, 1992). While CDRs guide antigen specificity, only a fraction of their residues directly contacts antigens (Dunbar et al., 2014), and numerous binding residues occur outside canonical CDRs (Kunik et al., 2012). Recent studies have demonstrated that representing paratope–epitope interactions using a standardized and compact vocabulary can improve the predictability of antibody–antigen binding from sequence data (Akbar et al., 2021). Predicting paratopes at residue resolution is therefore essential for antibody engineering, docking, repertoire profiling, and therapeutic design. Humanization (the process of genetically engineered non-human antibodies to minimize immunogenicity in humans while retaining their antigen-binding specificity) and modification of antibody frameworks can significantly influence paratope conformation and binding specificity (Almagro and Fransson, 2008). However, accurate prediction is challenging due to CDR loop flexibility, the subtlety of antigen–antibody interfaces, and strong class imbalance, where binding residues typically comprise ∼10% of the sequence (Berman et al., 2000). Structure-based techniques such as homology modeling and docking provide valuable insights (Sivasubramanian et al., 2009; Vreven et al., 2015) but rely on static templates and oversimplified scoring, limiting adaptability to dynamic interactions (Wodak et al., 2013).

Sequence-based approaches have advanced from early machine learning models, such as support vector machines and random forests, which relied on handcrafted physicochemical features (Ruffolo et al., 2022), to deep learning methods capable of modeling long-range dependencies (LeCun et al., 2015). Bidirectional long short-term memory (BiLSTM) networks (Hochreiter and Schmidhuber, 1997; Schuster and Paliwal, 1997; Siami-Namini et al., 2019) and convolutional neural networks (CNNs) have been successfully applied to capture sequence context and local motifs. Parapred (Liberis et al., 2018) combines CNNs and BiLSTMs using input windows consisting of CDRs plus two flanking residues on either side (CDR ± 2), while ParaAntiProt (Kalemati et al., 2024) integrates pretrained protein language models (PLMs) with CNNs. These methods have demonstrated good predictive power but often lack explicit chain-specific modeling and, in some cases, rely on predefined CDR segmentation, introducing variability from external annotation tools. Recent PLM-based models such as ESM-2 (Lin et al., 2023) and AntiBERTy (Leem et al., 2022) offer strong sequence representations but require substantial computational resources and can sacrifice model interpretability.

Structure-based approaches exploit three-dimensional information to improve accuracy. PECAN (Pittala and Bailey-Kellogg, 2020) uses graph neural networks (GNNs) with attention to capture context-aware structural representations, while Paragraph (Chinery et al., 2022) applies equivariant GNNs to antibody CDR ± 2 regions. ParaSurf (Papadopoulos et al., 2025) leverages 3D ResNet architectures with transformer-derived features to achieve state-of-the-art performance, but depends on Fab-region structures (the antigen-binding portion of antibody comprises VH, the first heavy chain constant domain; CH1, VL, and light chain constant domain; CL), limiting applicability when structural data are unavailable. Experimental mapping methods such as AbMap (Qi et al., 2021) provide high-throughput residue-level annotations but are restricted to linear epitopes. While structure-based methods can offer high spatial precision, they typically require high resolution or well-refined PDB structures to achieve accurate prediction, making them less practical for large-scale or early-stage discovery. A comparative summary of representative sequence-based, structure-based, and hybrid paratope prediction methods is presented in Table 1.

**TABLE 1:** Comparative summary of representative paratope prediction methods.

Model	Input type	Method	Chain-specificity	MCC range^a^	Strengths	Weaknesses
Liberis et al. (2018)	Sequence (CDR ± 2^b^)	CNN + BiLSTM	No	0.35–0.45	Efficient; sequence-only model	No chain awareness; limited context
Pittala and Bailey-Kellogg (2020)	Structure (Ab + Ag)	GNN + attention + transfer learning	No	0.55–0.65	Captures paired Ab-Ag interface	Needs both Ag and Ab structures
Chinery et al. (2022)	Structure (CDR ± 2, Ab only)	EGNN with minimal features	No	0.65–0.69	Antigen-agnostic; precise	Needs PDB input; CDR ± 2 only
Kalemati et al. (2024)	Sequence (Full chain or CDRs)	PLM embeddings + CNN	Partial	0.55–0.59	High accuracy; nanobody-capable	CDR-restricted input, limited interpretability

^a^ Ranges reflect reported Matthews correlation coefficients from respective publications; not all values are directly comparable due to dataset differences.

^b^ CDR ± 2 refers to complementarity-determining regions with two adjacent framework residues included at each boundary.

**FIGURE 1:**
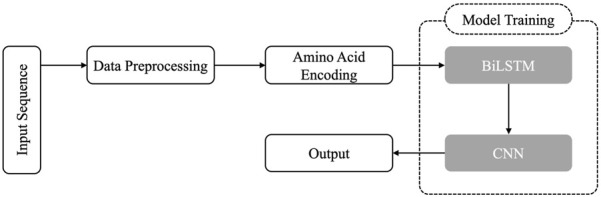
Framework for protein binding site prediction using Integrated BiLSTM-CNN Model.

Despite these advances, no prior study has systematically investigated chain-specific, sequence-only modeling for residue-level paratope prediction across a wide range of convolutional receptive fields and encoding strategies. To address this gap, we introduce ParaDeep, a chain-aware BiLSTM–CNN framework trained directly on full-length antibody sequences using either one-hot encoding or learnable embeddings. By combining BiLSTM layers to capture global sequence dependencies with CNN layers to detect local binding motifs, thereby balancing long-range contextual awareness with motif-level sensitivity. We systematically evaluated 30 model configurations varying in encoding schemes, kernel sizes, and antibody chain types (heavy (H), light (L), and combined heavy-light (HL)) using five-fold cross-validation, followed by independent blind test evaluation. Results demonstrate that chain-specific training markedly enhances predictive accuracy, particularly for heavy chains, and that kernel size is a critical determinant of performance. The overall ParaDeep framework is illustrated in Figure 1.

## 2 Materials and methods

### 2.1 Data preparation

#### 2.1.1 Dataset and chain annotation

A curated dataset of 2,807 antibody–antigen complexes was retrieved from the Antigen–Antibody Complex Database (AACDB; Zhou et al., 2025; https://i.uestc.edu.cn/AACDB/), version 1.0 (released 30 May 2024), accessed on 16 June 2025. The dataset contains paired heavy (H) and light (L) antibody chains for each complex, yielding a total of 5,614 sequences (2,807 H chains and 2,807 L chains). Binding residues were labeled using AACDB’s atom-distance method, which classifies an antibody residue as interacting (label = 1) if at least one atom in the residue is within the proximity range defined by AACDB’s atom-distance criterion to any atom in an antigen residue; otherwise, the residue is labeled as non-binding (label = 0).

To ensure the structural relevance and consistency of antibody variable domains in our analysis, we limited sequences to the typical length of antibody variable domains (VH and VL, approximately 110–130 residues), rather than an arbitrary cutoff based on a fixed number of initial residues or a specific numbering scheme (e.g., Chothia). This approach ensures that we include biologically relevant full variable regions while excluding constant domains or incomplete entries, which are not the focus of paratope prediction in this study.

Furthermore, the dataset was curated at the complex level. No additional redundancy reduction (e.g., sequence identity clustering) was applied to the sequences, as our aim was to capture the full diversity of VH and VL repertoires present in PDB-resolved complexes and assess our model’s performance across this natural variability. Certain PDB entries do not initiate residue numbering at position 1, indicating potential issues with structural completeness or annotation. By applying this length cutoff, we aimed to eliminate structurally inconsistent or biologically irrelevant antibody complexes, retaining only those suitable for meaningful paratope analysis and downstream modeling. Amino acids were represented either through one-hot encoding or learnable embeddings.

#### 2.1.2 Dataset statistics

**FIGURE 2:**
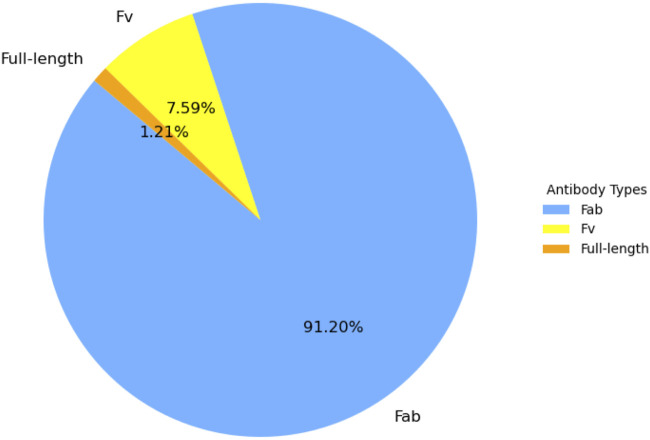
Distribution of antibody structural types in the dataset (n = 2,807 complexes).

The final dataset used for model development comprised 2,807 heavy (H) chains and 2,807 light (L) chains, yielding a total of 5,614 antibody sequences. Collectively, these sequences contained 716,896 residues, of which 74,350 (10.37%) were annotated as binding and 642,546 as non-binding, as defined in the AACDB. This distribution reflects a pronounced class imbalance, where binding residues constitute only ∼10% of the total, representing a typical challenge in supervised classification for protein–protein interaction prediction. In terms of structural formats, the dataset encompassed three main antibody types: Fab (n = 2,560; 91.20%), representing the antigen-binding fragment (VH + CH1 + VL + CL); Fv (n = 213; 7.59%), consisting only of the variable fragment (VH + VL); and full-length antibodies (n = 34; 1.21%), containing intact heavy and light chains. The distribution of antibody types is summarized in Table 2 and illustrated in Figure 2, where Fab fragments clearly dominate the dataset.

**TABLE 2:** Antibody structural type distribution in the dataset with corresponding frequencies, percentages, and descriptions.

Antibody type	Frequency	Percentage	Description
Fab	2,560	91.20%	Antigen-binding fragment (VH + CH1 + VL + CL)
Fv	213	7.59%	Variable fragment (VH + VL)
Full-length	34	1.21%	Intact heavy and light chain antibodies

#### 2.1.3 Amino acid representation

Two encoding schemes were applied to numerically represent amino acid sequences. In the one-hot encoding scheme, each residue was mapped to a 21-dimensional binary vector (representing the 20 standard amino acids plus an additional category for unknown residues, denoted as “X”), thereby preserving categorical relationships without introducing artificial ordinality (Mikolov et al., 2013).

In the learnable embedding scheme, input residues were first converted into integer indices ranging from 0 to 20, corresponding to the 20 standard amino acids and a special token for padding or unknown residues. These indices were then mapped to trainable dense vectors of dimension 21 using a PyTorch embedding layer. The embedding vectors were randomly initialized and optimized during training, allowing the network to learn context-specific representations of amino acids directly from sequence data (Asgari and Mofrad, 2015; Heffernan et al., 2016).

Both encoding methods were systematically evaluated across all model configurations to assess their effect on prediction performance.

#### 2.1.4 Chain-specific model design

To evaluate the effect of chain-aware learning, models were trained under three configurations: H-only (H), using heavy chain sequences exclusively; L-only (L), using light chain sequences exclusively; and HL-combined (HL), trained on a pooled dataset of both heavy and light chain sequences. This design enabled a systematic comparison between specialized (chain-specific) and generalized (combined) modeling approaches, allowing investigation into whether chain identity influences predictive performance. For HL-combined models, heavy and light chain sequences were not concatenated per antibody. Instead, H and L sequences were pooled into a single dataset and trained under one shared model architecture, with each input sequence (H or L) processed individually. This design allows the model to learn features common to both chain types without assuming direct inter-chain sequence dependency within a single input.

#### 2.1.5 Train–test split

To ensure robust model development and fair generalization assessment, the dataset was partitioned at the antibody–antigen complex level into a 90% modeling set and a 10% blind test set (222 complexes). The modeling set (2,585 complexes) was further split using five-fold stratified grouped cross-validation, ensuring that paired heavy and light chains from the same complex were assigned to the same fold to prevent information leakage.

### 2.2 Bidirectional long short-term memory (BiLSTM)

**FIGURE 3:**
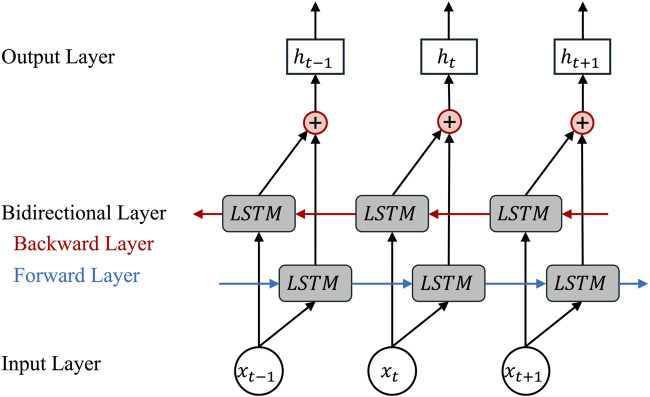
BiLSTM model architecture. The input sequence $\left[x_{t-1},x_{t},x_{t+1}\right]$ is processed simultaneously by a forward LSTM (blue arrows) and a backward LSTM (red arrows), producing hidden states in both directions. The outputs from the two directions are concatenated at each position to form the final bidirectional hidden state $h_{t}$ .

A Bidirectional Long Short-Term Memory (BiLSTM) network (Hochreiter and Schmidhuber, 1997; Schuster and Paliwal, 1997) extends the standard LSTM by processing sequences in both forward and reverse directions. This bidirectional context allows each residue representation to incorporate information from both upstream and downstream positions, which is particularly important for protein binding site prediction where interacting residues may be far apart in the primary sequence but close in three-dimensional space (Hanson et al., 2019; Liberis et al., 2018). The structural diagram of the BiLSTM module is shown in Figure 3.

Given an input sequence $X=\left[x_{1},x_{2},\ldots ,x_{T}\right]$ , where $x_{t}\in R^{d}$ is the residue feature vector at position t , the BiLSTM computes two hidden state sequences: the forward hidden states $\vec{h_{t}}$ and the backward hidden states $\overset{⃖}{h_{t}}$ . These are calculated as:

$$\vec{h_{t}}={LSTM}^{\to }\left(x_{t},\vec{h_{t-1}}\;\right)$$ (1)

$$\overset{⃖}{h_{t}}={LSTM}^{\leftarrow }\left(x_{t},\overset{⃖}{h_{t-1}}\right)$$ (2)

The final hidden $h_{t}$ representation at position t is obtained by concatenating the outputs from both directions:

$$h_{t}=\left[\vec{h_{t}}\;⨁\;\overset{⃖}{h_{t}}\right]\in R^{2H}$$ (3)

where ⨁ represents the concatenation symbol. H is the hidden size of each LSTM layer. This concatenated vector $h_{t}$ captures both long-range dependencies and bidirectional residue interactions, providing a richer sequence context for downstream convolutional layers to detect local paratope motifs.

### 2.3 Convolutional neural networks (CNN)

**FIGURE 4:**
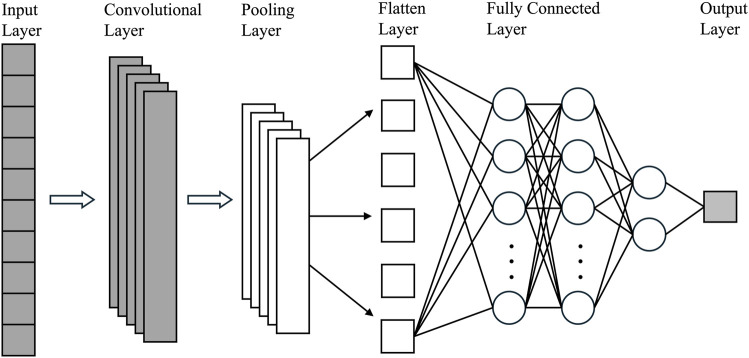
1D CNN model architecture. The input sequence is encoded and processed by a 1D convolutional layer with sliding kernels to generate feature maps that capture learned patterns. These features are then passed through a dense layer and sigmoid activation to yield per-position predictions.

Convolutional Neural Networks (CNNs) are well suited for detecting local patterns in structured data and have been widely applied to sequence-based bioinformatics problems, including protein–ligand and antibody–antigen binding site prediction (Liberis et al., 2018; Zeng et al., 2016; Alipanahi et al., 2015), building upon foundational work in gradient-based learning and convolutional architectures (LeCun et al., 1998). In this context, one-dimensional CNNs (1D CNNs) slide learnable filters along the sequence to extract motif-like features that may correspond to conserved biochemical interaction patterns, a concept similar to their application in text classification where convolutional filters capture local n-gram patterns (Kim, 2014). The structural diagram of the CNN module is shown in Figure 4.

Given an input $H=\left[h_{1},h_{2},\ldots ,h_{T}\right]$ , and a convolutional kernel of size k , the output at position t , denoted $z_{t}$ , is computed as:

$$z_{t}=\sum_{i=0}^{k-1}w_{i}\cdot h_{t+i}+b$$ (4)

where $w_{i}$ $\in R^{d}$ are the kernel weights and b is the bias term, and d is the input feature dimension. This output is passed through a non-linear activation function, typically ReLU:

$${\hat{z}}_{t}=\max\left(0,z_{t}\right)$$ (5)

Multiple convolutional kernels with different sizes are applied in parallel to capture patterns across varying sequence spans, from short local motifs to broader regions relevant for antigen recognition. Pooling operations, common in other domains, are omitted to preserve the per-residue spatial resolution necessary for paratope prediction. In ParaDeep, kernel sizes of 7, 15, 31, 71, and 130 residues were selected to represent short-, medium-, and long-range receptive fields along the antibody sequence. Smaller kernels enable the detection of compact, localized motifs, while larger kernels aggregate information from widely separated residues, which is important when paratope residues span multiple CDRs or extend into framework regions. This approach is conceptually analogous to the optimization of sliding window sizes in protein sequence and structure prediction (Chen et al., 2006), where window length critically determines the context available for feature extraction.

### 2.4 BiLSTM-CNN for protein binding site prediction

**FIGURE 5:**
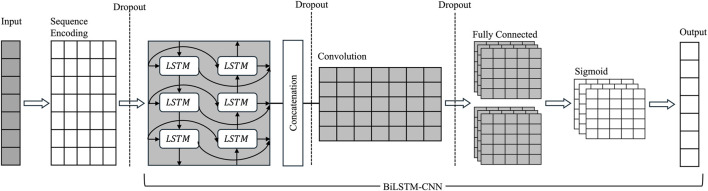
BiLSTM-CNN model architecture with dropout for residue-level binding site prediction. The input protein sequence of fixed length is first converted into one-hot or learnable embedding representations. A dropout layer is applied to the embeddings to reduce overfitting. The encoded sequence is then processed by a bidirectional LSTM (BiLSTM) layer to capture long-range contextual information, followed by another dropout layer on the BiLSTM output. The contextual features are transposed and passed through a 1D convolutional layer with a kernel of predefined size to capture local sequence patterns. Dropout is applied once more to the convolutional features before transposing back to sequence-aligned format. Finally, a fully connected layer maps each residue’s local features to a logit, and a sigmoid activation is applied to produce per-residue binding probabilities.

The BiLSTM–CNN module serves as the core prediction component in our protein binding site detection pipeline, integrating the operations formally described in Equations 4–9. It combines long-range contextual modeling via a Bidirectional Long Short-Term Memory (BiLSTM) network with local pattern extraction using one-dimensional Convolutional Neural Networks (1D CNN). This residue-level framework builds on prior work in deep learning-based motif recognition and antibody paratope prediction (Liberis et al., 2018; Ruffolo et al., 2022), which has shown that BiLSTM–CNN architectures can deliver strong predictive performance while maintaining interpretability in antibody–antigen interaction modeling. The overall architecture is shown in Figure 5 and summarized in Algorithm 1.

1. Sequence Encoding: Protein sequences are zero-padded to a fixed length of 130 residues to ensure uniform input dimensions. Each residue is encoded using one of two strategies:
  • One-hot encoding: a binary vector of length 21 (20 amino acids + unknown residue ‘X’).
  • Learnable embedding: a trainable vector of dimension $x_{t}\in R^{21}$

2. Contextualization *via* BiLSTM: The encoded sequence is processed by a BiLSTM layer with a hidden size of 64 per direction, yielding a contextual vector $h_{t}\in R^{128}$ at each position, as defined in Equations 1–3. The BiLSTM is well-suited for capturing long-range dependencies in protein sequences where binding residues may be far apart in sequence but close in three-dimensional space (Hanson et al., 2019; Liberis et al., 2018).
3. Local Feature Extraction with 1D CNN: The BiLSTM output is transposed and processed by a 1D CNN using multiple kernel sizes $k\in \left\{7,15,31,71,\text{Full}\right\}$ . Each convolutional operation generates local feature maps:
$$z_{t}=f\left(\sum_{i=0}^{k-1}w_{i}\cdot h_{t+i}+b\right)$$ (6)

where $f\left(\cdot \right)$ is the ReLU, $w_{i}$ are kernel weights, and $z_{t}\in R^{H}$ captures local sequence motifs (Liberis et al., 2018).

4. Per-Residue Prediction: Each local feature vector $z_{t}$ is passed through a fully connected layer:
$$y_{t}=W\cdot z_{t}+b$$ (7)

followed by a sigmoid activation to obtain the predicted binding probability:

$$p_{t}=\frac{1}{1+e^{-y_{t}}}\in \left[0,1\right]$$ (8)
  where W and b are learnable parameters.
5. Regularization via Dropout: Dropout ( p=0.3 ) is applied after the embedding layer, BiLSTM output, and CNN feature maps to reduce overfitting by preventing co-adaptation of hidden units.
6. Handling Class Imbalance: To address the imbalance between binding and non-binding residues, the model uses a weighted binary cross-entropy loss (He and Garcia, 2009), a strategy conceptually similar to cost-sensitive learning approaches previously applied in protein-binding site prediction (Wu and Zhou, 2017):
$$L=-\sum_{t=1}^{L}\left[{w_{+}y}_{t}\log\left(p_{t}\right)+w_{-}\left({1-y}_{t}\right)\log\left(1-p_{t}\right)\right]$$ (9)

where $w_{+}=\frac{N_{neg}}{N_{pos}}$ is weight for positive class. $N_{neg}$ and $N_{pos}$ denote the number of negative and positive labels, respectively. The negative class weight is implicitly set to $w_{-}=1$ . For the final model configuration, we used optimized weights of $w_{-}=1$ and $w_{+}=8.616$ , derived from the ratio of non-binding to binding residues in the training set. This weighting scheme penalizes errors on the minority (binding) class more strongly and encourages the model to assign higher importance to correctly identifying binding residues. This method is supported by prior work on class imbalance (He and Garcia, 2009; Liberis, et al., 2018) and implemented in deep learning frameworks like PyTorch (Paszke, et al., 2019), with masking applied to ignore padded residues during training.

7. Optimization and Training: Training is performed using the Adam optimizer with a learning rate of 0.001 (Paszke et al., 2019). Padding masks are applied to exclude padded residues from loss computation. Early stopping is used to prevent overfitting, where training is terminated if the validation loss does not improve for 5 consecutive epochs. This strategy promotes better generalization and reduces the risk of overtraining.

Algorithm 1 Pseudo-code of the BiLSTM-CNN binding site prediction model.

Input: Amino-acid sequence S (padded to length L=130 ).
Output: per-residue binding probabilities ${\left\{p_{t}\right\}}_{t=1}^{L}$

  1. Residue Encoding
   Map each residue $s_{t}$ to $x_{t}\in R^{d}$ using either one-hot encoding ( d=21 ) or a learnable embedding ( d=21 ).

$$X=\left[x_{1},x_{2},\ldots ,x_{L}\right]\in R^{L\times d}$$

  2. Dropout (Embedding)
   Apply dropout to the embedding representation to prevent overfitting.

$$X\leftarrow Dropout\left(X,p=0.3\right)$$

  3. BiLSTM Contextualization
   Pass the embedded input into a bidirectional LSTM layer to obtain contextual representations.
      − Forward hidden state: $\vec{h_{t}}={LSTM}^{\to }\left(x_{t},\vec{h_{t-1}}\;\right)$

      − Backward hidden state: $\overset{⃖}{h_{t}}={LSTM}^{\leftarrow }\left(x_{t},\overset{⃖}{h_{t-1}}\right)$

      − Concatenate: $h_{t}=\left[\vec{h_{t}}\;⨁\;\overset{⃖}{h_{t}}\right]\in R^{2H}$ (with H=64 )
      − Collect: $H^{\left(seq\right)}=\left[h_{1},\ldots ,h_{L}\right]\in R^{L\times 2H}$

  4. Dropout (BiLSTM)
   Apply dropout to the BiLSTM output to enhance generalization.

$$H^{\left(seq\right)}\leftarrow Dropout\left(H^{\left(seq\right)},p=0.3\right)$$

  5. 1D Convolution without Pooling
   Reshape BiLSTM output to match CNN input shape.
   Transpose for CNN: $H^{\left(cnn\_in\right)}\in R^{2H\times L}$

   For each kernel size $k\in \left\{7,15,31,71,L\right\}$ (use same padding to preserve length L ):

$$z_{t}^{\left(k\right)}=\text{ReLU}\left(\sum_{i=0}^{k-1}w_{i}^{\left(k\right)}\cdot h_{t+i}+b^{\left(k\right)}\right)$$

   Concatenate feature maps over kernels to obtain $Z\in R^{L\times C}$

    ( C is the total number of convolutional filters across all kernel sizes.)
  6. Dropout (CNN)
   Apply dropout to the CNN output to reduce over-reliance on specific features:

$$Z\leftarrow Dropout\left(Z,p=0.3\right)$$

  7. Per-residue classifier
   Logit: $y_{t}=W\cdot z_{t}+b$

   Probability: $p_{t}=\sigma \left(y_{t}\right)=1/\left(1+e^{{-y}_{t}}\right)$

  8. Masked, class-weighted binary cross-entropy
   Positive weight: $w_{+}=\frac{N_{neg}}{N_{pos}}$ , negative weight $w_{-}=1$

   Loss (mask padded positions):

$$L=-\sum_{t=1}^{L}m_{t}\left[w_{+}y_{t}^{*}\log\left(p_{t}\right)+w_{-}\left(1-y_{t}^{*}\right)\log\left(1-p_{t}\right)\right]$$

   where $y_{t}^{*}\in \left\{0,1\right\}$ and $m_{t}\in \left\{0,1\right\}$ is the padding mask (1 = real residue, 0 = padding).

  9. Optimization and Early Stopping: Train the model using the Adam optimizer with a learning rate of 0.001; terminate training if validation loss fails to improve for 5 consecutive epochs.

### 2.5 Training procedure and experimental design

The BiLSTM–CNN architecture was trained and evaluated using a five-fold cross-validation protocol on the training set of 2,585 antibody–antigen complexes. In each fold, 80% of the data were used for training and 20% for validation, with stratification at the complex level to ensure paired heavy (H) and light (L) chains from the same complex were not split across sets. Performance metrics were averaged across folds and reported as mean ± standard deviation. In addition to the general model trained on all chain types, chain-specific models were developed for H-only, L-only, and HL-combined configurations using the same cross-validation scheme. Each chain-specific model was evaluated exclusively on its corresponding chain type to assess the influence of chain identity on predictive performance. Generalization was further tested on an independent blind hold-out set of 222 complexes, withheld from all training and hyperparameter tuning stages. These complexes included residue-level binding site annotations, enabling rigorous, unbiased evaluation on structurally diverse and previously unseen samples.

### 2.6 Model evaluation and performance metrics

Model performance was assessed on the independent blind test set using both threshold-dependent and threshold-independent metrics. From the confusion matrix, we computed the standard classification metrics:

$$\text{Precision}=\frac{TP}{TP+FP}$$

$$\text{Recall}=\frac{TP}{TP+FN}$$

$$F1=\frac{2\cdot Precision\cdot Recall}{Precision+Recall}$$

$$\text{Accuracy}=\frac{TP+TN}{TP+TN+FP+FN}$$

$$\text{BAC}=\frac{1}{2}\left(\frac{TP}{\text{TP}+\text{FN}}+\frac{TN}{TN+FP}\right)$$

$$\text{MCC}=\frac{TP\cdot TN-FP\cdot FN}{\sqrt{\left(TP+FP\right)\left(TP+FN\right)\left(TN+FP\right)\left(TN+FN\right)}}$$

Here, true positives (TP) and true negatives (TN) refer to correctly predicted binding and non-binding residues, respectively, while false positives (FP) and false negatives (FN) indicate incorrect predictions. We also report the area under the receiver operating characteristic curve (AUC-ROC) and the area under the precision–recall curve (PR AUC). Importantly, to address the class imbalance inherent in paratope datasets, we emphasize the use of F1-score, Balanced Accuracy (BAC), and Matthews Correlation Coefficient (MCC), all of which are well-established and robust metrics for evaluating binary classifiers under skewed class distributions. PR AUC offers a more informative evaluation under severe class imbalance by focusing on the trade-off between precision and recall (Davis and Goadrich, 2006), which is particularly relevant for binding site prediction where positive residues typically constitute a small fraction of the sequence. This perspective aligns with the unified framework for analyzing performance measures proposed by Wu and Zhou (2013), which emphasizes selecting metrics appropriate to the prediction setting.

## 3 Results

### 3.1 Overview of model configurations

To systematically investigate residue-level paratope prediction, we developed 30 BiLSTM–CNN model configurations by varying three primary factors: (i) amino acid encoding scheme (one-hot vs. learnable embedding), (ii) antibody chain type (heavy (H), light (L), and combined (HL)), and (iii) convolutional kernel size (7, 15, 31, 71, and 130 residues). All sequences were zero-padded to a uniform length of 130 residues. Models were trained using five-fold cross-validation with MCC-based early stopping (patience = 5 epochs) to select the best epoch per fold. The complete configuration set is detailed in Supplementary Table S1. For downstream benchmarking against the sequence-based baseline Parapred, we selected four representative ParaDeep models (M1–M4; Table 3). These representatives were chosen to capture the best-performing configurations for each chain type and encoding strategy, enabling both within-chain and cross-chain performance comparisons on the blind test set.

**TABLE 3:** Representative chain-specific BiLSTM-CNN models evaluated on the blind test set. All models were trained using five-fold cross-validation with MCC-based early stopping (patience = 5 epochs).

Model id	Encoding	Chain	Kernel size	Description
M1	Embedding	H	130	Best H-chain embedding model
M2	One-hot	H	130	Best H-chain one-hot model
M3	Embedding	L	130	Best L-chain embedding model
M4	One-hot	L	130	Best L-chain one-hot model

### 3.2 Comparison of encoding strategies

We evaluated the impact of encoding strategies on model performance by comparing F1 and Matthews Correlation Coefficient (MCC) across H, L, and HL chains with varying convolutional kernel sizes.

**FIGURE 6:**
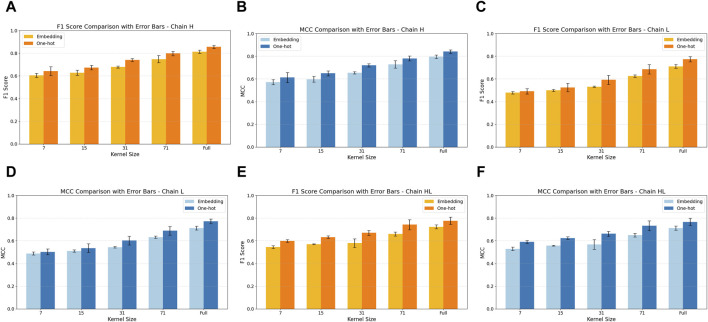
Comparison of F1 and Matthews Correlation Coefficient (MCC) between embedding-based and one-hot encoding strategies across various convolutional kernel sizes, evaluated on HL chain data. Panels **(A,B)** present results for H chain models; **(C,D)** for L chain models; and **(E,F)** for HL chain models. Each bar represents the mean performance over five-fold cross-validation, with error bars indicating the mean ± standard deviation.

For H-chain models, the one-hot encoded configuration achieved the highest performance, with an F1 of 0.856 ± 0.014 and MCC of 0.842 ± 0.015 at full kernel size (130 residues). In comparison, the embedding-based model achieved F1 = 0.813 ± 0.015 and MCC = 0.796 ± 0.016 (Figures 6A,B; Supplementary Table S2).

A similar pattern was observed for L-chain models (Figures 6C,D). The one-hot model achieved a peak F1 of 0.774 ± 0.023 and MCC of 0.772 ± 0.022, outperforming the embedding-based counterpart (F1 = 0.711 ± 0.017, MCC = 0.712 ± 0.016) at the same kernel size (Supplementary Table S3). While absolute performance for L chains was lower than for H chains, the relative superiority of one-hot encoding was consistent across all kernel sizes.

For HL-chain models, the same trend persisted (Figures 6E,F). The best one-hot model achieved F1 of 0.777 ± 0.031 and MCC of 0.767 ± 0.031, compared to F1 = 0.723 ± 0.017 and MCC = 0.712 ± 0.018 for the embedding-based model (Supplementary Table S4). Notably, the performance gap between encodings schemes widened with increasing kernel size, suggesting that one-hot encoding benefits more from broader sequence context than learnable embeddings.

While one-hot encoding outperformed embeddings in terms of F1 and MCC across most configurations, embedding-based models achieved slightly higher Balanced Accuracy (BAC), with only marginal differences between the two approaches (Supplementary Table S7). Together, these results underscore the robustness and efficiency of one-hot encoding for residue-level paratope prediction. Despite lacking trainable parameters, one-hot vectors consistently outperformed learned embeddings across all chain types and kernel sizes, particularly in models with wide convolutional receptive fields.

### 3.3 Impact of convolutional kernel size

**FIGURE 7:**
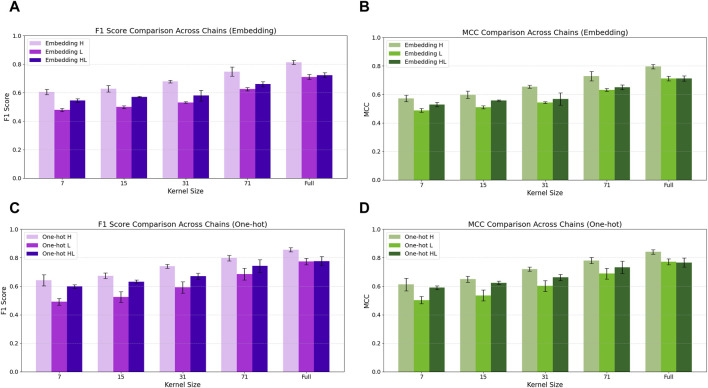
Comparison of F1 (mean ± standard deviation) and Matthews Correlation Coefficient (MCC) across convolutional kernel sizes for embedding-based and one-hot encoded models trained on H, L, and HL chain datasets. Subfigures **(A,B)** display the F1 and MCC for embedding models, while **(C,D)** show the corresponding metrics for one-hot models. All models were trained with early stopping and evaluated on a shared test set comprising H, L, and HL chains. Each bar represents the average performance across five-fold cross-validation, with error bars indicating ± one standard deviation.

We systematically evaluated the impact of convolutional kernel size on model performance across encoding strategies (embedding vs. one-hot) and antibody chain types (H, L, HL). As illustrated in Figure 7, increasing the kernel size from 7 to the full sequence length (130 residues) consistently improved both F1 and Matthews Correlation Coefficient (MCC) across all model configurations.

For embedding-based models (Figures 7A,B), performance increased steadily with kernel size, although absolute metrics remained lower than those of one-hot models. On the H chain, F1 improved from 0.605 ± 0.019 (kernel size 7) to 0.813 ± 0.015 (full length), with MCC rising from 0.572 ± 0.023 to 0.796 ± 0.016. L and HL chain models showed similar trends, reaching maximum values of F1 ≈ 0.711–0.723 and MCC ≈0.712, but consistently lagged behind the one-hot counterparts (Supplementary Table S5). Balanced Accuracy (BAC) values for embedding-based models showed small but consistent increases across kernel sizes, supporting the modest gains observed in F1 and MCC (Supplementary Table S8).

In one-hot encoded models (Figures 7C,D), performance gains with larger kernels were pronounced. For the H chain, F1 and MCC increased from 0.642 ± 0.039 and 0.613 ± 0.044 (kernel = 7) to 0.856 ± 0.014 and 0.842 ± 0.015 (full length), respectively. L chain models improved from F1 = 0.491 ± 0.024, MCC = 0.503 ± 0.025 to F1 = 0.774 ± 0.023, MCC = 0.772 ± 0.022, while HL models peaked at F1 = 0.777 ± 0.031 and MCC = 0.767 ± 0.031 (see Supplementary Table S6). One-hot encoded models exhibited slight BAC improvements with larger kernels, although the differences were relatively small (Supplementary Table S9).

Interestingly, intermediate kernel sizes—particularly kernel size 71—offered near-peak performance with reduced computational cost. For example, the one-hot H chain model with kernel 71 attained F1 = 0.799 ± 0.020 and MCC = 0.781 ± 0.021, closely approximating the full-length results. These results highlight the importance of kernel width in capturing long-range residue dependencies in paratope prediction. While full-length kernels yield the highest accuracy, mid-sized kernels offer a practical balance between performance and efficiency, making them well-suited for deployment in resource-constrained environments.

### 3.4 Impact of chain-specific modeling

**FIGURE 8:**
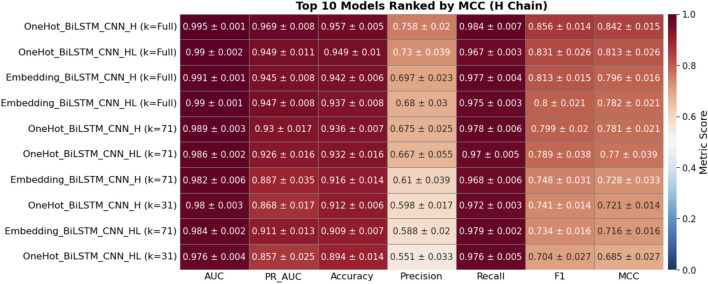
Heatmap showing the performance metrics of the top 10 deep learning models on the H chain validation dataset, based on five-fold cross-validation. Each cell presents the mean ± standard deviation for key evaluation metrics, including AUC, PR AUC, Accuracy, Precision, Recall, F1, and Matthews Correlation Coefficient (MCC). Models are ranked in descending order by MCC to emphasize those with superior binding site prediction performance. The color intensity corresponds to the magnitude of each metric, with red tones indicating higher values and blue tones indicating lower values.

**FIGURE 9:**
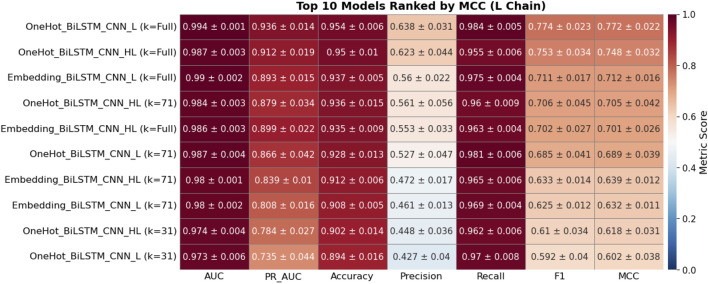
Heatmap showing the performance metrics of the top 10 deep learning models on the L chain validation dataset, based on five-fold cross-validation. Each cell presents the mean ± standard deviation for key evaluation metrics, including AUC, PR AUC, Accuracy, Precision, Recall, F1, and Matthews Correlation Coefficient (MCC). Models are ranked in descending order by MCC to emphasize those with superior binding site prediction performance. The color intensity corresponds to the magnitude of each metric, with red tones indicating higher values and blue tones indicating lower values.

**FIGURE 10:**
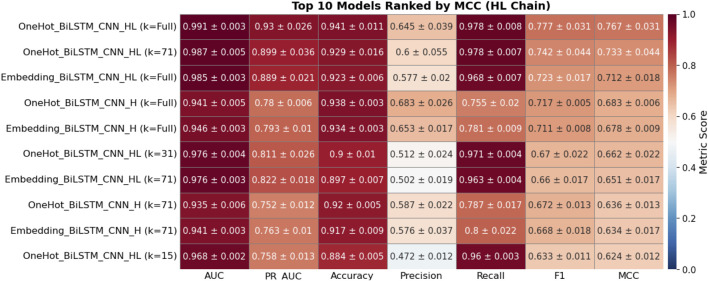
Heatmap showing the performance metrics of the top 10 deep learning models on the HL chain validation dataset, based on five-fold cross-validation. Each cell presents the mean ± standard deviation for key evaluation metrics, including AUC, PR AUC, Accuracy, Precision, Recall, F1, and Matthews Correlation Coefficient (MCC). Models are ranked in descending order by MCC to emphasize those with superior binding site prediction performance. The color intensity corresponds to the magnitude of each metric, with red tones indicating higher values and blue tones indicating lower values.

To investigate the importance of chain specialization, 30 BiLSTM-CNN configurations were evaluated on heavy (H), light (L), and combined heavy–light (HL) chain validation sets. Models were ranked by mean Matthews Correlation Coefficient (MCC) from five-fold cross-validation, with the top 10 for each dataset shown in Figures 8–10.

**FIGURE 11:**
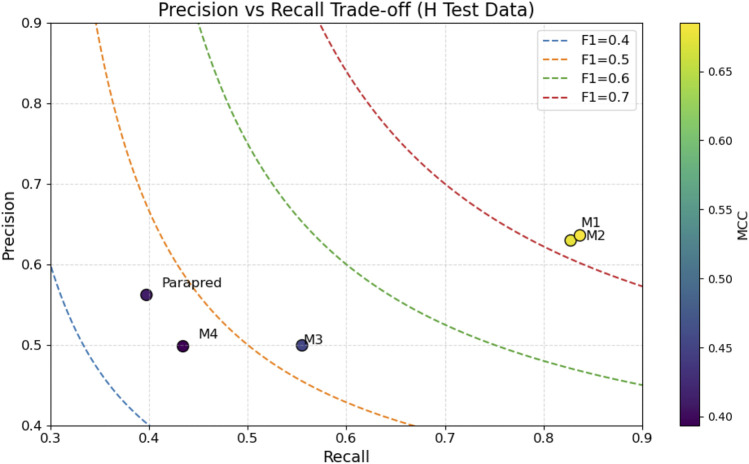
Precision–Recall Trade-off for Heavy Chain Models. The plot compares Parapred and proposed models M1–M4 on the H chain blind test set, with F1 iso-contours and MCC color gradient.

On the H-chain validation set (Figure 8), models trained exclusively on H-chain sequences consistently outperformed L- or HL-trained models. The best-performing configuration—one-hot encoding with a full-length kernel—achieved F1 = 0.856 ± 0.014 and MCC = 0.842 ± 0.015. Notably, all top 10 models in this category were H-trained, reflecting the strong predictive signal in heavy chains.

For the L-chain validation set (Figure 9), the top model was also chain-specific (one-hot encoding, full-length kernel), reaching F1 = 0.774 ± 0.023 and MCC = 0.772 ± 0.022. Although some HL-trained models appeared in the top 10, they consistently underperformed compared to L-specific models.

On the HL-chain test set (Figure 10), the highest-ranked model was trained on HL sequences and achieved F1 = 0.777 ± 0.031, MCC = 0.767 ± 0.031. However, this score still fell slightly below the best H-chain model tested on H-chain data, suggesting that mixed-chain training may dilute chain-specific features essential for high-precision binding site prediction.

Across all three validation sets, one-hot encoding outperformed embedding-based models. The top embedding models recorded MCC values between 0.712 and 0.796, consistently below their one-hot counterparts. These results highlight the advantages of chain-specific modeling, particularly for the heavy chain, which demonstrated robust and consistent predictive power. Based on these findings, we prioritized H- and L-chain models for blind test evaluation and excluded HL-trained models from downstream benchmarking.

### 3.5 Comparison with existing method on the blind test dataset

On the blind test set comprising 222 antibody–antigen complexes, the proposed ParaDeep models (M1–M4) consistently outperformed the sequence-based baseline, Parapred, across all key evaluation metrics. These results demonstrate the strong generalization capability of the chain-aware BiLSTM–CNN framework and highlight the benefits of full-length convolution and one-hot encoding for capturing long-range sequence dependencies in residue-level paratope prediction. The four representative ParaDeep configurations (Table 3) were selected as the top-performing models from five-fold cross-validation: two trained on heavy (H) chains (M1–M2) and two on light (L) chains (M3–M4), each using either one-hot or embedding-based encoding. Models were evaluated separately on the H- and L-chain subsets of the blind test set, alongside Parapred.

Heavy chain evaluation (Table 4) showed that the embedding-based H-chain model (M1) achieved the highest overall performance, with AUC = 0.959, PR AUC = 0.805, F1 = 0.723, and MCC = 0.685. Its one-hot counterpart (M2) also performed strongly (F1 = 0.715, MCC = 0.676). In contrast, L-trained models (M3–M4) showed markedly lower MCC values (<0.460) when evaluated on H-chain sequences, underscoring the importance of chain-specific training. Parapred scored an MCC of 0.410, substantially below both M1 and M2.

**TABLE 4:** Performance of ParaDeep models and Parapred on the heavy-chain blind test set. The best value for each metric is shown in bold.

Model Id	Description	AUC	PR AUC	Accuracy	Precision	Recall	F1	MCC
M1	BiLSTM–CNN, embedding, H	**0.959**	**0.805**	**0.919**	**0.636**	**0.837**	**0.723**	**0.685**
M2	BiLSTM–CNN, one-hot, H	0.956	0.790	0.917	0.630	0.827	0.715	0.676
M3	BiLSTM–CNN, embedding, L	0.866	0.509	0.873	0.499	0.555	0.526	0.454
M4	BiLSTM–CNN, one-hot, L	0.838	0.459	0.873	0.498	0.434	0.464	0.394
Parapred	Parapred (baseline)	0.861	0.516	0.884	0.562	0.397	0.466	0.410

Light chain evaluation (Table 5) reversed the trend: the one-hot L-chain model (M4) achieved the best performance, with F1 = 0.607 and MCC = 0.587, followed closely by the embedding-based L-chain model (M3). H-trained models (M1–M2) underperformed in this setting, further confirming the chain specificity of learned features. Parapred again lagged behind, with F1 = 0.437 and MCC = 0.404.

**TABLE 5:** Performance of ParaDeep models and Parapred on the light-chain blind test set. The best value for each metric is shown in bold.

Model id	Description	AUC	PR AUC	Accuracy	Precision	Recall	F1	MCC
M1	BiLSTM–CNN, embedding, H	0.862	0.391	0.915	0.432	0.467	0.449	0.403
M2	BiLSTM–CNN, one-hot, H	0.855	0.388	0.914	0.423	0.473	0.447	0.401
M3	BiLSTM–CNN, embedding, L	0.948	**0.708**	0.913	0.451	**0.828**	0.584	0.571
M4	BiLSTM–CNN, one-hot, L	**0.945**	0.697	**0.925**	**0.495**	0.786	**0.607**	**0.587**
Parapred	Parapred (baseline)	0.861	0.442	0.928	0.509	0.383	0.437	0.404

**FIGURE 12:**
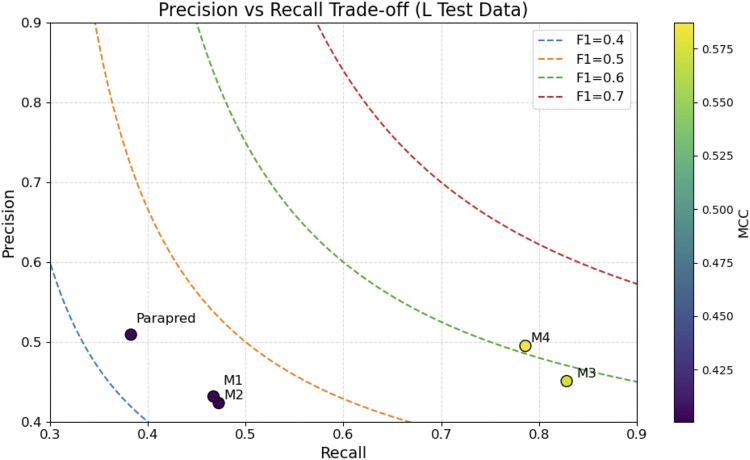
Precision–Recall Trade-off for Heavy Chain Models. The plot compares Parapred and proposed models M1–M4 on the L chain blind test set, with F1 iso-contours and MCC color gradient.

**FIGURE 13:**
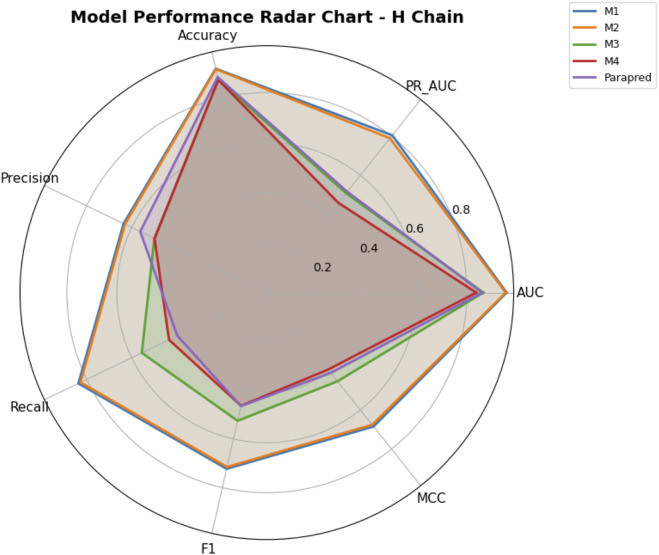
Radar Chart of Model Performance on Heavy Chain. Summary of metrics (AUC, PR AUC, Accuracy, Precision, Recall, F1, MCC) for each model on the H chain blind test set.

**FIGURE 14:**
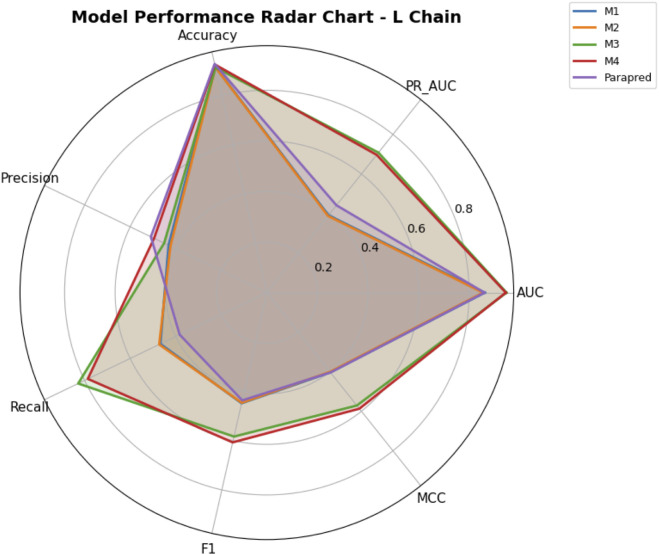
Radar Chart of Model Performance on Light Chain. Summary of metrics (AUC, PR AUC, Accuracy, Precision, Recall, F1, MCC) for each model on the L chain blind test set.

Precision–recall trade-offs are illustrated in Figures 11, 12 for H- and L-chain evaluations, respectively, with F1 contours and MCC color encoding. Figures 13, 14 present radar plots comparing all models across seven metrics (AUC, PR AUC, accuracy, precision, recall, F1, and MCC). Overall, ParaDeep’s chain-aware BiLSTM–CNN models deliver consistent improvements over existing sequence-based approaches, particularly in metrics robust to class imbalance, such as PR AUC and MCC. These findings reinforce the value of chain-specific modeling for high-fidelity paratope prediction.

## 4 Discussion

### 4.1 Summary of key findings

This study introduced ParaDeep, a sequence-based deep learning framework for residue-level paratope prediction that integrates bidirectional long short-term memory (BiLSTM) networks with one-dimensional convolutional neural networks (CNNs). BiLSTM layers capture bidirectional sequence dependencies (Hochreiter and Schmidhuber, 1997; Schuster and Paliwal, 1997), while CNN layers extract local structural motifs relevant to binding (Liberis et al., 2018; Hanson et al., 2019). We systematically evaluated 30 model configurations by varying three primary factors: amino acid encoding strategy (one-hot vs. learnable embedding), antibody chain type (H, L, and HL), and convolutional kernel size. The results demonstrated that chain-specific training, long-range convolution, and appropriate encoding strategy are critical determinants of model performance, in line with prior evidence that chain-aware modeling enhances antibody binding site prediction (Ruffolo et al., 2022). On a blind test set of 222 antibody–antigen complexes, ParaDeep outperformed the widely used sequence-based baseline Parapred (Liberis et al., 2018) across all key metrics, including precision–recall AUC and Matthews correlation coefficient, which are particularly informative under class imbalance. This generalization capability, achieved without requiring structural input, underscores ParaDeep’s potential as a scalable, structure-independent tool for early-stage antibody design, complementing structure-based methods such as AlphaFold (Jumper et al., 2021) or graph-based approaches like ParaAntiProt (Kalemati et al., 2024).

While attention mechanisms and transformer-based architectures are well-known for their ability to capture long-range dependencies and offer interpretability, our initial focus in this study was to prioritize model interpretability through the analysis of learned motifs and to maintain lightweight deployment capabilities. We designed our architecture to demonstrate the effectiveness of combining BiLSTM and CNN layers for residue-level paratope prediction. Future work will benchmark attention layers and full transformer-based architectures against our current model to assess potential gains in performance, computational efficiency, and enhanced interpretability, providing a comprehensive comparison of different mechanisms for capturing sequence context.

### 4.2 Chain-dependent effects of encoding strategy

While one-hot encoding yielded superior results in most cross-validation settings, chain-specific blind test evaluations revealed a more nuanced pattern. On the H-chain dataset, the embedding-based model (M1) slightly outperformed its one-hot counterpart (M2). Biologically, this advantage is likely driven by the higher sequence and structural diversity of heavy chains, particularly in the CDR-H3 region, which exhibits the greatest variability in length, amino acid composition, and conformational flexibility among antibody loops (Xu and Davis, 2000; Kuroda et al., 2012). Such diversity provides a rich feature space for learnable embeddings to capture subtle biochemical similarities and contextual dependencies, beyond what discrete one-hot representations can offer. This is consistent with the principle that embeddings project residues into a continuous vector space, enabling proximity-based relationships between amino acids (Mikolov et al., 2013; Peters et al., 2018).

In contrast, for the L-chain dataset, the one-hot encoded model (M4) outperformed the embedding-based model (M3). Light chains are generally more conserved in sequence and structure, with reduced loop variability compared to heavy chains (Almagro et al., 2019; Abhinandan and Martin, 2008). Computationally, one-hot encoding avoids additional trainable parameters, reducing the risk of overfitting when sequence diversity is low (Goodfellow et al., 2016). Sparse categorical encodings also preserve exact residue identity, which can be advantageous when modeling conserved motifs (Krizhevsky et al., 2017; Wu et al., 2021).

Our model architecture consistently performed better on antibody heavy chains (VH) compared to light chains (VL). While heavy chains are known for their higher sequence and structural diversity, this variability, paradoxically, can provide richer and more distinct signals for learnable embeddings to capture contextual dependencies. The increased information content within VH sequences, especially concerning CDR H3, which is the most diverse and often central to antigen binding, allows the model’s embedding layers to learn more discriminative features. Thus, this ‘diversity’ enhances the model’s ability to learn meaningful representations rather than inherently hindering it, particularly when coupled with architectures capable of capturing complex patterns from these richer signals. Light chains, being less diverse, might offer fewer distinct features for the model to leverage, leading to slightly lower performance.

From a computational perspective, these results underscore that encoding strategy should align with both biological diversity and dataset characteristics. Embeddings can leverage variability in diverse repertoires such as H chains, while one-hot encoding remains a robust choice for conserved repertoires like L chains. This observation aligns with findings from protein language modeling studies, where encoding choices directly influence downstream task performance (Elnaggar et al., 2022; Rao et al., 2019).

### 4.3 Effect of convolutional kernel size

Convolutional kernel size was a critical determinant of ParaDeep’s predictive performance. Across all encoding strategies and antibody chain types, models employing full-length convolution (kernel = 130 residues) achieved the highest F1 and MCC scores. This improvement reflects the biological reality that antibody paratopes can span multiple complementarity-determining regions (CDRs) and may also include framework residues (Saha and Raghava, 2006; Chen, Kurgan, and Ruan, 2008; Jones, 1999). Such residues are often distant in the primary sequence yet spatially close in three-dimensional space, cooperating to form the antigen-binding interface (Sela-Culang et al., 2013).

The superior performance of ‘full-length convolution’ (which is structurally analogous to a fully connected layer applied across the entire sequence) over standard CNN filters indicates that paratope residues are not solely determined by short-range local flanking residues. Instead, paratope residues often span multiple CDRs and framework regions, requiring a broader, more global sequence context for accurate prediction. This result does not indicate a failure of CNNs, but highlights that their effectiveness in this task depends strongly on the kernel size, which determines the accessible sequence context. The biological distribution of binding residues across disparate segments of the variable domain necessitates a model that can capture these long-range dependencies effectively.

From a computational standpoint, larger convolutional kernels expand the receptive field of the CNN, allowing aggregation of features over broad sequence contexts. This capability complements the BiLSTM’s bidirectional context modeling by enabling the detection of distributed motifs that span multiple structural segments. As discussed by Araujo et al. (2019), the size of the receptive field is directly related to kernel width and network depth, with larger receptive fields providing the ability to capture global sequence patterns.

However, large kernels also increase the number of trainable parameters and computational cost per forward pass, which can impact scalability in large-scale applications. Interestingly, our results revealed that mid-sized kernels (e.g., 71 residues) achieved near-peak performance while significantly reducing computation. This balance aligns with the bias–variance trade-off described by Goodfellow et al. (2016), in which overly large models risk overfitting, while excessively small kernels may underfit. Furthermore, findings from Gehring et al. (2017) in convolutional sequence modeling show that intermediate receptive fields can capture most relevant dependencies without incurring the computational and overfitting risks of full-length kernels.

Overall, kernel size tuning emerges as both a biologically and computationally significant hyperparameter in paratope prediction. While full-length kernels maximize performance by capturing all possible long-range dependencies, mid-sized kernels provide an attractive trade-off between accuracy and efficiency, making them particularly suitable for deployment in real-time or resource-constrained antibody design workflows.

### 4.4 Comparison with prior methods

ParaDeep consistently outperformed Parapred (Liberis et al., 2018), a widely adopted sequence-based paratope predictor, in both H- and L-chain blind test evaluations. On the H-chain set, ParaDeep achieved a relative MCC improvement of over 27%, with corresponding gains in F1 and PR AUC. On the L-chain, ParaDeep similarly outperformed Parapred across all metrics. These improvements can be attributed to ParaDeep’s chain-specific modeling, class imbalance handling, and deep contextual architecture. Parapred employs a CNN–BiLSTM architecture but does not incorporate chain-specific training, instead using a single model for all antibody chains. This lack of specialization, coupled with its shorter convolutional kernels, limits its ability to capture long-range dependencies critical for high-fidelity paratope prediction.

ParaSurf represents a leading structure-based approach for paratope prediction. The most recent version, ParaSurf (Papadopoulos et al., 2025), integrates surface geometric, chemical, and force-field features using a hybrid 3D ResNet and transformer architecture. This method achieves state-of-the-art results on multiple benchmark datasets, including prediction across the entire Fab region. However, ParaSurf’s reliance on high-quality 3D antibody structures limits its utility in early-stage antibody discovery pipelines, where structural data may be incomplete or unavailable. Although structure prediction tools such as AlphaFold (Jumper et al., 2021) can mitigate this requirement, they introduce additional computational overhead and modeling uncertainty. ParaAntiProt (Kalemati et al., 2024) offers another deep learning–based sequence predictor, but it requires explicit CDR segmentation during both training and inference. This dependency introduces variability due to differences in numbering schemes (Dunbar and Deane, 2016) and definitions of CDR boundaries (Chothia and Lesk, 1987), potentially affecting reproducibility across datasets and studies.

In contrast, ParaDeep operates directly on raw amino acid sequences without requiring structural input or domain-specific segmentation. This design choice enables fair and reproducible comparisons across datasets, isolates the benefits of the BiLSTM–CNN architecture from preprocessing biases, and makes the method adaptable to varied antibody formats and discovery pipelines. For benchmarking, Parapred was chosen as the primary sequence-based comparator, as it shares ParaDeep’s input modality and preprocessing simplicity, allowing a direct assessment of architectural improvements.

### 4.5 Practical implications and limitations

ParaDeep is well-suited for high-throughput antibody discovery, particularly in early-stage workflows where structural information is unavailable. Its reliance solely on primary amino acid sequences enables application to antibodies without resolved 3D structures, making it ideal for computational pre-screening prior to experimental validation. The modular architecture and compact parameter count (<10 M) allow efficient deployment on standard computing resources without specialized hardware, supporting both academic and industrial use. In hybrid pipelines, ParaDeep can be paired with structural modeling tools such as AlphaFold (Jumper et al., 2021) and docking platforms like ClusPro (Kozakov et al., 2017) to refine downstream structural analyses, acting as a rapid sequence-based filter to narrow candidates before more computationally intensive modeling.

Nevertheless, limitations remain. First, the training dataset primarily comprises canonical Fab and Fv formats, leaving generalization to single-chain variable fragments (scFv), nanobodies, and synthetic constructs untested. Second, although sequence-based methods such as ParaDeep capture biochemical and contextual relationships between residues, they may lack the atomic-level spatial precision achievable by structure-based methods (Krawczyk et al., 2013). Third, while weighted binary cross-entropy loss mitigates class imbalance (Buda et al., 2018; Wu and Zhou, 2017), rare paratope configurations may still be underrepresented, potentially biasing predictions toward more common binding patterns.

Future improvements include expanding training data to encompass scFv, nanobody, and synthetic constructs; integrating attention mechanisms or graph-based encoders (Gligorijević et al., 2021) to enhance spatial reasoning without full 3D inputs; and exploring pretrained protein language models such as ESM-2 (Lin et al., 2023) or AntiBERTy (Ruffolo et al., 2021) to enrich residue embeddings while maintaining interpretability.

### 4.6 Computational techniques and design considerations

Building on these practical insights, ParaDeep’s BiLSTM–CNN framework was designed to balance interpretability, accuracy, and computational efficiency. Bidirectional LSTMs capture long-range dependencies in both sequence directions (Hochreiter and Schmidhuber, 1997), while 1D CNNs serve as adaptive context windows for detecting local binding motifs. Kernel sizes were optimized per chain type to reflect biological variation in paratope patterns, with mid-to long-range kernels (71 and 130 residues) delivering the best results.

Unlike pretrained protein language models (PLMs) such as ESM-2 (Lin et al., 2023) or AntiBERTy (Ruffolo et al., 2021)—which offer high representational power but demand significant computational resources and can reduce transparency (Choromanski et al., 2020; Kalemati et al., 2024)—ParaDeep uses lightweight, task-specific embeddings, enabling fast, resource-efficient deployment.

To address severe class imbalance between binding and non-binding residues, weighted binary cross-entropy loss was applied, a method shown effective in prior studies (Buda et al., 2018; Wu and Zhou, 2017). An ablation study of 30 model variants revealed that early stopping had minimal impact, likely due to stable training from class weighting.

Overall, ParaDeep’s hybrid architecture achieves chain-aware, interpretable modeling without relying on structural input or PLMs. This makes it a practical and flexible alternative to structure-dependent or PLM-heavy predictors (Kalemati et al., 2024; Liberis et al., 2018), suitable for diverse paratope prediction scenarios.

## 5 Conclusion

This study introduces ParaDeep, a lightweight and interpretable deep learning framework for residue-level paratope prediction, based solely on amino acid sequences. The proposed chain-aware BiLSTM-CNN architecture integrates bidirectional context modeling with adaptive 1D convolution, enabling the extraction of both local and non-local sequence features without reliance on structural input or pretrained embeddings.

Across 30 systematically evaluated model configurations, embedding-based models generally outperformed one-hot encodings, particularly on heavy chains, while chain-specific training led to the highest overall performance—especially for light chains, which benefited from longer receptive fields. Optimal kernel sizes varied by chain, reflecting distinct biological binding characteristics.

Compared to structure-based tools like ParaSurf, ParaDeep achieved comparable performance on key metrics (e.g., MCC, F1), while offering significantly lower computational overhead and broader usability in sequence-only contexts. Importantly, ParaDeep outperformed the sequence-based baseline Parapred on the same benchmark dataset. These results highlight ParaDeep’s strength in capturing functional antibody features in a structure-independent and resource-efficient manner.

With its generalization across chain types, sensitivity to CDR3 diversity, and ability to detect binding signals in non-CDR regions, ParaDeep represents a practical tool for early-stage antibody discovery, repertoire profiling, and therapeutic design—particularly under conditions where structural data is unavailable or incomplete.

## Data Availability

The original contributions presented in this study are included in the article and Supplementary Material. The dataset analyzed in this study is available at the Antigen–Antibody Complex Database (AACDB; Zhou et al., 2025) https://i.uestc.edu.cn/AACDB/.
