# Supplementary material for: ParaDeep: sequence-based deep learning for residue-level paratope prediction using chain-aware BiLSTM-CNN models
Source: Front Bioinform. 2025 Nov 5;5:1684042. doi: 10.3389/fbinf.2025.1684042 (PMC12626946; doi:10.3389/fbinf.2025.1684042)
Supplement: Supplementary file 1 [file Supplementaryfile1.docx]

**Table S1*.*** Summary of All BiLSTM-CNN Model Configurations Evaluated in This Study
This table presents the complete set of 30 BiLSTM-CNN model configurations, detailing each combination of encoding strategy (one-hot or embedding), antibody chain type (H, L, HL), and convolutional kernel size used during training.

| **Model ID** | **Encoding Scheme** | **Antibody Chain** | **Kernel Size** |
| --- | --- | --- | --- |
| **1** | One-hot | H | 7 |
| **2** | One-hot | H | 15 |
| **3** | One-hot | H | 31 |
| **4** | One-hot | H | 71 |
| **5** | One-hot | H | Full |
| **6** | One-hot | L | 7 |
| **7** | One-hot | L | 15 |
| **8** | One-hot | L | 31 |
| **9** | One-hot | L | 71 |
| **10** | One-hot | L | Full |
| **11** | One-hot | HL | 7 |
| **12** | One-hot | HL | 15 |
| **13** | One-hot | HL | 31 |
| **14** | One-hot | HL | 71 |
| **15** | One-hot | HL | Full |
| **16** | Embedding | H | 7 |
| **17** | Embedding | H | 15 |
| **18** | Embedding | H | 31 |
| **19** | Embedding | H | 71 |
| **20** | Embedding | H | Full |
| **21** | Embedding | L | 7 |
| **22** | Embedding | L | 15 |
| **23** | Embedding | L | 31 |
| **24** | Embedding | L | 71 |
| **25** | Embedding | L | Full |
| **26** | Embedding | HL | 7 |
| **27** | Embedding | HL | 15 |
| **28** | Embedding | HL | 31 |
| **29** | Embedding | HL | 71 |
| **30** | Embedding | HL | Full |

### ****Table S2.** Performance Comparison of One-hot and Embedding Models for H Chain Across Kernel Sizes**

This table presents a comparative analysis of F1 Score and Matthews Correlation Coefficient (MCC) for one-hot and embedding-based BiLSTM-CNN models trained on the H chain dataset. Models were evaluated across various convolutional kernel sizes (7, 15, 31, 71, and full length) using five-fold cross-validation. Each value represents the mean performance, with ± indicating one standard deviation.

#### ****Table S2a.** F1 Score (mean ± std) for H Chain Models**

| **Kernel Size** | **Embedding** | **One-hot** |
| --- | --- | --- |
| 7 | 0.605 ± 0.019 | 0.642 ± 0.039 |
| 15 | 0.628 ± 0.023 | 0.674 ± 0.020 |
| 31 | 0.679 ± 0.009 | 0.741 ± 0.014 |
| 71 | 0.748 ± 0.031 | 0.799 ± 0.020 |
| Full | 0.813 ± 0.015 | 0.856 ± 0.014 |

#### ****Table S2b.** Matthews Correlation Coefficient (MCC) (mean ± std) for H Chain Models**

| **Kernel Size** | **Embedding** | **One-hot** |
| --- | --- | --- |
| 7 | 0.572 ± 0.023 | 0.613 ± 0.044 |
| 15 | 0.597 ± 0.026 | 0.650 ± 0.022 |
| 31 | 0.655 ± 0.010 | 0.721 ± 0.014 |
| 71 | 0.728 ± 0.033 | 0.781 ± 0.021 |
| Full | 0.796 ± 0.016 | 0.842 ± 0.015 |

### ****Table S3.** Performance Comparison of One-hot and Embedding Models for L Chain Across Kernel Sizes**

This table reports the F1 Score and Matthews Correlation Coefficient (MCC) of BiLSTM-CNN models trained on the L chain dataset using embedding and one-hot encoding strategies. Model performance was evaluated across increasing convolutional kernel sizes using five-fold cross-validation. Results are shown as mean ± standard deviation.

#### ****Table S3a.** F1 Score (mean ± std) for L Chain Models**

| **Kernel Size** | **Embedding** | **One-hot** |
| --- | --- | --- |
| 7 | 0.479 ± 0.011 | 0.491 ± 0.024 |
| 15 | 0.500 ± 0.009 | 0.525 ± 0.038 |
| 31 | 0.532 ± 0.006 | 0.592 ± 0.040 |
| 71 | 0.625 ± 0.012 | 0.685 ± 0.041 |
| Full | 0.711 ± 0.017 | 0.774 ± 0.023 |

#### ****Table S3b.** Matthews Correlation Coefficient (MCC) (mean ± std) for L Chain Models**

| **Kernel Size** | **Embedding** | **One-hot** |
| --- | --- | --- |
| 7 | 0.488 ± 0.013 | 0.503 ± 0.025 |
| 15 | 0.511 ± 0.010 | 0.536 ± 0.038 |
| 31 | 0.543 ± 0.006 | 0.602 ± 0.038 |
| 71 | 0.632 ± 0.011 | 0.689 ± 0.039 |
| Full | 0.712 ± 0.016 | 0.772 ± 0.022 |

### ****Table S4.** Performance Comparison of One-hot and Embedding Models for HL Chain Across Kernel Sizes**

This table presents the F1 Score and Matthews Correlation Coefficient (MCC) for BiLSTM-CNN models trained on combined H and L chain (HL) data. Both one-hot and embedding encoding strategies were evaluated across five convolutional kernel sizes. Reported values represent the mean ± standard deviation over five-fold cross-validation.

#### ****Table S4a.** F1 Score (mean ± std) for HL Chain Models**

| **Kernel Size** | **Embedding** | **One-hot** |
| --- | --- | --- |
| 7 | 0.545 ± 0.013 | 0.600 ± 0.013 |
| 15 | 0.570 ± 0.004 | 0.633 ± 0.011 |
| 31 | 0.580 ± 0.038 | 0.670 ± 0.022 |
| 71 | 0.660 ± 0.017 | 0.742 ± 0.044 |
| Full | 0.723 ± 0.017 | 0.777 ± 0.031 |

#### ****Table S4b.** Matthews Correlation Coefficient (MCC) (mean ± std) for HL Chain Models**

| **Kernel Size** | **Embedding** | **One-hot** |
| --- | --- | --- |
| 7 | 0.530 ± 0.015 | 0.590 ± 0.013 |
| 15 | 0.558 ± 0.005 | 0.624 ± 0.012 |
| 31 | 0.567 ± 0.043 | 0.662 ± 0.022 |
| 71 | 0.651 ± 0.017 | 0.733 ± 0.044 |
| Full | 0.712 ± 0.018 | 0.767 ± 0.031 |

### ****Table S5.** Detailed Performance Metrics (F1 Score and MCC) of Embedding Models Across Kernel Sizes for H, L, and HL Chains**

This table provides a comprehensive summary of the performance of embedding-based BiLSTM-CNN models across varying convolutional kernel sizes for H, L, and HL chains. Metrics include the F1 Score and Matthews Correlation Coefficient (MCC), reported as the mean ± standard deviation over five-fold cross-validation.

#### ****Table S5a.** F1 Score (mean ± std) for Embedding Models**

| **Kernel Size** | **H Chain** | **L Chain** | **HL Chain** |
| --- | --- | --- | --- |
| 7 | 0.605 ± 0.019 | 0.479 ± 0.011 | 0.545 ± 0.013 |
| 15 | 0.628 ± 0.023 | 0.500 ± 0.009 | 0.570 ± 0.004 |
| 31 | 0.679 ± 0.009 | 0.532 ± 0.006 | 0.580 ± 0.038 |
| 71 | 0.748 ± 0.031 | 0.625 ± 0.012 | 0.660 ± 0.017 |
| Full | 0.813 ± 0.015 | 0.711 ± 0.017 | 0.723 ± 0.017 |

#### ****Table S5b.** Matthews Correlation Coefficient (MCC) (mean ± std) for Embedding Models**

| **Kernel Size** | **H Chain** | **L Chain** | **HL Chain** |
| --- | --- | --- | --- |
| 7 | 0.572 ± 0.023 | 0.488 ± 0.013 | 0.530 ± 0.015 |
| 15 | 0.597 ± 0.026 | 0.511 ± 0.010 | 0.558 ± 0.005 |
| 31 | 0.655 ± 0.010 | 0.543 ± 0.006 | 0.567 ± 0.043 |
| 71 | 0.728 ± 0.033 | 0.632 ± 0.011 | 0.651 ± 0.017 |
| Full | 0.796 ± 0.016 | 0.712 ± 0.016 | 0.712 ± 0.018 |

### ****Table S6.** Detailed Performance Metrics (F1 Score and MCC) of One-hot Models Across Kernel Sizes for H, L, and HL Chains**

This table summarizes the performance of one-hot encoded BiLSTM-CNN models trained on H, L, and HL chains across five convolutional kernel sizes. Metrics include F1 Score and Matthews Correlation Coefficient (MCC), reported as the mean ± standard deviation over five-fold cross-validation.

#### ****Table S6a.** F1 Score (mean ± std) for One-hot Models**

| **Kernel Size** | **H Chain** | **L Chain** | **HL Chain** |
| --- | --- | --- | --- |
| 7 | 0.642 ± 0.039 | 0.491 ± 0.024 | 0.600 ± 0.013 |
| 15 | 0.674 ± 0.020 | 0.525 ± 0.038 | 0.633 ± 0.011 |
| 31 | 0.741 ± 0.014 | 0.592 ± 0.040 | 0.670 ± 0.022 |
| 71 | 0.799 ± 0.020 | 0.685 ± 0.041 | 0.742 ± 0.044 |
| Full | 0.856 ± 0.014 | 0.774 ± 0.023 | 0.777 ± 0.031 |

#### ****Table S6b.** Matthews Correlation Coefficient (MCC) (mean ± std) for One-hot Models**

| **Kernel Size** | **H Chain** | **L Chain** | **HL Chain** |
| --- | --- | --- | --- |
| 7 | 0.613 ± 0.044 | 0.503 ± 0.025 | 0.590 ± 0.013 |
| 15 | 0.650 ± 0.022 | 0.536 ± 0.038 | 0.624 ± 0.012 |
| 31 | 0.721 ± 0.014 | 0.602 ± 0.038 | 0.662 ± 0.022 |
| 71 | 0.781 ± 0.021 | 0.689 ± 0.039 | 0.733 ± 0.044 |
| Full | 0.842 ± 0.015 | 0.772 ± 0.022 | 0.767 ± 0.031 |

### ****Table S7.**** Balanced Accuracy (BAC) of One-hot and Embedding Models for H, L, and HL Chains Across All Kernel Sizes.

This table presents a comparative summary of Balanced Accuracy (BAC) for BiLSTM-CNN models using one-hot and embedding encodings, trained on heavy (H), light (L), and combined (HL) antibody chains. Models were evaluated across five convolutional kernel sizes (7, 15, 31, 71, and full length) using five-fold cross-validation. Values are reported as mean ± standard deviation.

#### ****Table S7a.** Balanced Accuracy (BAC) (mean ± std) for H Chain Models**

| **Kernel Size** | **Embedding** | **One-hot** |
| --- | --- | --- |
| 7 | 0.841 ± 0.008 | 0.849 ± 0.002 |
| 15 | 0.854 ± 0.014 | 0.847 ± 0.006 |
| 31 | 0.855 ± 0.016 | 0.849 ± 0.010 |
| 71 | 0.879 ± 0.011 | 0.859 ± 0.006 |
| Full | 0.886 ± 0.002 | 0.862 ± 0.010 |

#### ****Table S7b.** Balanced Accuracy (BAC) (mean ± std) for L Chain Models**

| **Kernel Size** | **Embedding** | **One-hot** |
| --- | --- | --- |
| 7 | 0.852 ± 0.005 | 0.844 ± 0.003 |
| 15 | 0.856 ± 0.005 | 0.843 ± 0.014 |
| 31 | 0.852 ± 0.014 | 0.844 ± 0.007 |
| 71 | 0.866 ± 0.009 | 0.854 ± 0.008 |
| Full | 0.867 ± 0.012 | 0.856 ± 0.009 |

#### ****Table S7c.** Balanced Accuracy (BAC) (mean ± std) for HL Chain Models**

| **Kernel Size** | **Embedding** | **One-hot** |
| --- | --- | --- |
| 7 | 0.850 ± 0.008 | 0.855 ± 0.005 |
| 15 | 0.851 ± 0.008 | 0.851 ± 0.007 |
| 31 | 0.864 ± 0.012 | 0.852 ± 0.010 |
| 71 | 0.869 ± 0.010 | 0.863 ± 0.006 |
| Full | 0.879 ± 0.006 | 0.86 ± 0.022 |

### ****Table S8.**** Balanced Accuracy (BAC) of Embedding-based Models Across Kernel Sizes for H, L, and HL Chains.

This table reports the Balanced Accuracy (BAC) of BiLSTM-CNN models using embedding-based amino acid representations, evaluated separately on H, L, and HL chains across five kernel sizes. Results are averaged over five-fold cross-validation and expressed as mean ± standard deviation.

#### ****Table S8.** Balanced Accuracy (BAC) (mean ± std) for Embedding Models**

| **Kernel Size** | **H Chain** | **L Chain** | **HL Chain** |
| --- | --- | --- | --- |
| 7 | 0.841 ± 0.008 | 0.852 ± 0.005 | 0.850 ± 0.008 |
| 15 | 0.854 ± 0.014 | 0.856 ± 0.005 | 0.851 ± 0.008 |
| 31 | 0.855 ± 0.016 | 0.852 ± 0.014 | 0.864 ± 0.012 |
| 71 | 0.879 ± 0.011 | 0.866 ± 0.009 | 0.869 ± 0.010 |
| Full | 0.886 ± 0.002 | 0.867 ± 0.012 | - 1. 0.006 |

### ****Table S9.**** Balanced Accuracy (BAC) of One-hot Encoded Models Across Kernel Sizes for H, L, and HL Chains.

This table summarizes the Balanced Accuracy (BAC) achieved by BiLSTM-CNN models using one-hot encoding, trained and evaluated on H, L, and HL chains across different convolutional kernel sizes. Each metric reflects the mean ± standard deviation from five-fold cross-validation.

#### ****Table S9.** Balanced Accuracy (BAC) (mean ± std) for One-hot Models**

| **Kernel Size** | **H Chain** | **L Chain** | **HL Chain** |
| --- | --- | --- | --- |
| 7 | 0.849 ± 0.002 | 0.844 ± 0.003 | 0.855 ± 0.005 |
| 15 | 0.847 ± 0.006 | 0.843 ± 0.014 | 0.851 ± 0.007 |
| 31 | 0.849 ± 0.010 | 0.844 ± 0.007 | 0.852 ± 0.010 |
| 71 | 0.859 ± 0.006 | 0.854 ± 0.008 | 0.863 ± 0.006 |
| Full | 0.862 ± 0.010 | 0.856 ± 0.009 | 0.860 ± 0.022 |

**Data Availability**

The original contributions presented in this study are included in the article and Supplementary Material. The dataset analyzed in this study is available at the Antigen–Antibody Complex Database (AACDB; Zhou et al., 2025) https://i.uestc.edu.cn/AACDB/.
